# Leveraging Deep Learning for Practical DoA Estimation: Experiments with Real Data Collected via USRP

**DOI:** 10.3390/s22197578

**Published:** 2022-10-06

**Authors:** Hyeonjin Chung, Hyunwoo Park, Sunwoo Kim

**Affiliations:** Department of Electronics and Computer Engineering, Hanyang University, Seoul 04763, Korea

**Keywords:** deep learning, direction-of-arrival estimation, deep neural network, convolutional neural network, universal software radio peripheral

## Abstract

This paper presents an experimental validation of deep learning-based direction-of-arrival (DoA) estimation by using realistic data collected via universal software radio peripheral (USRP). Deep neural network (DNN) and convolutional neural network (CNN) structures are designed to estimate the DoA. Two types of data are used for training networks. One is the data synthesized by the signal model, and the other is the data collected by USRP. Here, the signal model considers both mutual coupling and multipath signals. Experimental results show that the estimation performance is most accurate when training DNN and CNN with the collected data. Furthermore, the estimation tends to be poor in the indoor environment, which suffers from the strong non-line-of-sight (NLoS) signals.

## 1. Introduction

Direction-of-arrival (DoA) estimation is one of the long-studied research topics in array signal processing. DoA estimation algorithms have been adopted in various applications, such as localization and radar [1]. Traditional DoA estimation algorithms such as MUSIC [2] and ESPRIT [3] are proposed based on the characteristic of the signal model. Although they can ideally achieve high estimation accuracy and resolution, unexpected problems (e.g., multipath effect [4], gain-phase error [5], mutual coupling [6], antenna misalignment [7], etc.) may exist in practice. In this case, the signal model cannot capture the characteristics of a real received signal, thereby causing degradation of the DoA estimation performance.

There have been various studies to deal with problems that may cause model mismatch. One of these studies is coherent DoA estimation, which can estimate multipath signals impinging from different directions [4,8,9]. On the other hand, there are problems induced by hardware impairments such as gain-phase error, mutual coupling, and antenna misalignment. There have been efforts to calibrate these errors without using reference signals, where [5,10,11] deal with gain-phase error, [6,12,13] deal with mutual coupling between antennas in an array, and [7,14] deal with the errors in the steering vector that can be caused by antenna misalignment. However, the performance of the aforementioned works may degrade when several problems simultaneously occur or there are more unexpected problems.

After the introduction of deep learning [15], DoA estimation algorithms based on various types of neural network (NN) have been proposed in [16,17,18,19,20]. One of the benefits of using deep learning is that the user does not have to know the exact signal model if there are sufficient training data. For this reason, it is expected that the deep learning-based DoA estimation does not suffer from model mismatch problems by using data that capture all kinds of errors (e.g., actual measured data). In [16,17], deep neural network (DNN)-based DoA estimation was proposed, where these works report that the DNN-based estimation is more accurate than the traditional DoA estimation. After the proposal of the DNN-based DoA estimation, convolutional neural network (CNN)-based DoA estimation has been studied in [18,19]. The CNN-based DoA estimation shows better estimation accuracy and resolution compared to the DNN-based DoA estimation. In [20], the DoA estimation based on unsupervised learning was proposed, where unsupervised learning can make data collection easier since data labeling is not required. In recent works, there have been efforts to exploit features of classical DoA estimation, rather than solely depending on NN. Refs. [21,22,23] respectively employ DNN, CNN, and recurrent neural network (RNN) to estimate the ideal noiseless covariance matrix, which is denoted as a pseudo covariance matrix. Then, the classical DoA estimation such as MUSIC and root-MUSIC estimates the DoA with pseudo covariance matrix. In [24], the residual neural network (ResNet) first estimates the candidates of DoAs. From the candidates, the classical maximum likelihood estimation (MLE) [25] picks the final DoAs. A combination of these two methods enhances the accuracy while achieving lower complexity than only using MLE.

However, the existing works on the deep learning-based DoA estimation lack experimental validation, even though the deep learning-based DoA estimation is expected to be effective in a practical situation where there are many problems that cause a model mismatch. In this paper, we validate deep learning-based DoA estimation with realistic data collected by a universal software radio peripheral (USRP). In the experiment, two types of data—data synthesized by the signal model and data collected by USRP—are used for training networks. The estimation accuracy is then analyzed according to the type of training data.

## 2. System Model

In this paper, we consider one transmitter, which is equipped with an omni-directional antenna. A receiver is equipped with a uniform linear array (ULA), which has *M* antenna elements. The spacing between adjacent antennas is set to half-wavelength λ/2, where λ denotes the wavelength of the transmitted signal.

To generate the data for training DNN and CNN, the signal model should be defined. Here, the generated data are expected to be well-suited for training if the signal model can capture the state of the hardware systems. Among the many kinds of hardware-induced problems, the gain-phase error in our systems is calibrated using the method in [26]. The antenna spacing is designed to be half-wavelength so that there is no antenna misalignment. However, the current systems cannot calibrate mutual coupling and multipath effects. Thus, in this paper, we consider mutual coupling and multipath effects to design the received signal model.

An array manifold vector whose DoA is θ, a(θ) can be given as follows:

(1) $$a\left(\theta \right)={\left[1,e^{j\pi \cos\theta },\ldots ,e^{j(M-1)\pi \cos\theta }\right]}^{T}\in C^{M\times 1}.$$

To capture mutual coupling and multipath effects, we model a received signal $X\in C^{M\times D}$ as:

(2) $$X=C\sum_{p=0}^{P}\alpha_{p}a\left(\theta_{p}\right)s^{T}+N\in C^{M\times D}.$$

where $C\in C^{M\times M}$ denotes the mutual coupling matrix [27]. *P* denotes the number of non-line-of-sight (NLoS) paths. $\alpha_{p}$ and $\theta_{p}$ respectively denote the channel gain and the DoA of the *p*-th signal path. Specifically, $\alpha_{0}$ and $\theta_{0}$ denote the channel gain and the DoA of the line-of-sight (LoS) path. $s={\left[s_{1},\ldots ,s_{D}\right]}^{T}\in C^{D\times 1}$ denotes a signal vector, whose power equals $\sigma_{s}^{2}$. *D* is a number of signal snapshots. $N\in C^{M\times D}$ is a noise matrix, whose entries all follow $\mathrm{CN}(0,\sigma^{2})$. $\sigma^{2}$ denotes the power of the noise. $R_{X}$, the covariance matrix of X, can be defined as:

(3) $$\begin{array}{c} R_{X}=E\left[XX^{H}\right]\approx \frac{XX^{H}}{D}\in C^{M\times M}. \end{array}$$

## 3. Deep Learning Network Structure for DoA Estimation

**Figure 1:**
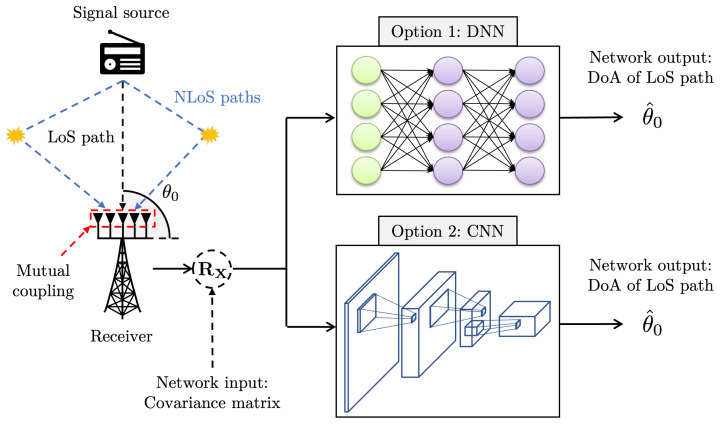
A scheme of deep learning-based DoA estimation. The proposed DNN or CNN structure estimates the DoA of the LoS path in the presence of multipath signals and mutual coupling.

This section introduces two network structures for DoA estimation, which are respectively based on DNN and CNN. A scheme of deep learning-based DoA estimation is depicted in Figure 1. In the presence of multipath signals and mutual couplings, the deep learning network aims to estimate the DoA of the LoS path using the covariance matrix.

### 3.1. DoA Estimation via Deep Neural Network

Since the input of the DNN has to be a real vector, the input of the DNN $\chi_{\mathrm{DNN}}\in R^{2M^{2}\times 1}$ is formulated as:

(4) $$\chi_{\mathrm{DNN}}=\mathrm{vec}\left(\left[\mathrm{real}\left(\frac{R_{X}}{{∥R_{X}∥}_{F}}\right),\mathrm{imag}\left(\frac{R_{X}}{{∥R_{X}∥}_{F}}\right)\right]\right),$$

where vec(·) denotes the vectorization. real(·) and imag(·) respectively denote the real and imaginary values of the argument. ${∥\cdot ∥}_{F}$ denotes the Frobenius norm.

Letting *L*, $d^{\left(l\right)}$, and $I^{\left(l\right)}$ respectively denote the number of dense layers, the output of the *l*-th layer, and the size of $d^{\left(l\right)}$, $d^{\left(l\right)}\left(j\right)$ can be given by:

(5) $$\begin{array}{cc} d^{\left(l\right)}\left(j\right)=\mathrm{ReLU}\left(\sum_{i=1}^{I^{\left(l-1\right)}}U^{\left(l\right)}\left(j,i\right)d^{\left(l-1\right)}\left(j\right)+v^{\left(l\right)}\left(j\right)\right), & \\ l=1,\ldots ,L, \end{array}$$

where $d^{\left(0\right)}=\chi_{\mathrm{DNN}}$. ReLU(·) denotes the ReLU function, which is widely used as the activation function of the neuron [15]. $U^{\left(l\right)}$ and $v^{\left(l\right)}$ denote the weights and the bias of the *l*-th layers. The loss function of the DNN is given by MSE, ${\left(\hat{\theta_{0}}-\theta_{0}\right)}^{2}$, where $\hat{\theta_{0}}$ denotes the estimated value of the DoA of the LoS path. The weights and the bias that minimize the loss function can be denoted as:

(6) $${\left\{{\hat{U}}^{\left(l\right)},{\hat{v}}^{\left(l\right)}\right\}}_{l=1}^{L}=\underset{{\left\{U^{\left(l\right)},v^{\left(l\right)}\right\}}_{l=1}^{L}}{\mathrm{argmin}}{\left(\hat{\theta_{0}}-\theta_{0}\right)}^{2},$$

where ${\hat{U}}^{\left(l\right)}$ and ${\hat{v}}^{\left(l\right)}$ denote the weights and the bias of the *l*-th dense layer that minimize the loss function. (6) is implemented by back propagation. A parameter setting for the DNN structure used in this paper is summarized in Table 1.

### 3.2. DoA Estimation via Convolutional Neural Network

Since the input of the CNN can be a three-dimensional matrix, the input of the CNN $\chi_{\mathrm{CNN}}\in R^{M\times M\times 2}$ is formulated as:

(7) $$\chi_{\mathrm{CNN}}=\left[\mathrm{real}\left(\frac{R_{X}}{{∥R_{X}∥}_{F}}\right);\mathrm{imag}\left(\frac{R_{X}}{{∥R_{X}∥}_{F}}\right)\right],$$

where ; denotes an operator that overlaps matrices with the same dimension.

An output of the *k*-th convolutional layer, $C^{\left(k\right)}$, can be represented as in [18]:

(8) $$\begin{array}{cc} C^{\left(k\right)}\left(:,:,j\right)=\mathrm{ReLU}\left(W_{j}^{\left(k\right)}*C^{\left(k-1\right)}+B_{j}^{\left(k\right)}\right), & \\ \mathrm{for}\;j=1,\ldots ,J(k),\;k=1,\ldots ,K, \end{array}$$

where ∗ denotes the convolution. $C^{\left(k\right)}\left(:,:,j\right)$ denotes the *j*-th channel of 3D tensor $C^{\left(k\right)}$ and $C^{\left(0\right)}=\chi_{\mathrm{CNN}}$. J(k) and *K* are the number of kernels and the number of convolutional layers. $W_{j}^{\left(k\right)}\in R^{Q_{k}\times Q_{k}}$ denotes the *j*-th kernel in the *k*-th layer, where $Q_{k}$ is a dimension of the kernels in the *k*-th layer. $B_{j}^{\left(k\right)}$ denotes the bias for the *j*-th kernel in the *k*-th convolutional layer.

After undergoing *K* convolutional layers, all values of $C^{\left(K\right)}$ are summed to yield the output. The loss function of the CNN is given by MSE, ${\left(\hat{\theta_{0}}-\theta_{0}\right)}^{2}$. The convolution kernel and the bias that minimize the loss function can be given by:

(9) $$\left\{{\hat{W}}_{j}^{\left(k\right)},{\hat{B}}_{j}^{\left(k\right)}\right\}=\underset{\left\{W_{j}^{\left(k\right)},B_{j}^{\left(k\right)}\right\}}{\mathrm{argmin}}{\left(\hat{\theta_{0}}-\theta_{0}\right)}^{2},\;\mathrm{for}\;j=1,\ldots ,J\left(k\right),\;k=1,\ldots ,K,$$

where ${\hat{W}}_{j}^{\left(k\right)}$ and ${\hat{B}}_{j}^{\left(k\right)}$ denote the *j*-th convolution kernel and the bias of the *k*-th layer that minimize the loss function. (9) is implemented by back propagation. A parameter setting for CNN structure used in this paper is summarized in Table 2.

## 4. Experimental Results and Discussion

### 4.1. Experimental Setup

In this paper, we use two types of data. One is synthesized data generated based on the signal model in (2). We generated 4,000,000 synthesized data via MATLAB. Here, *M*, *D*, and the maximum mutual coupling strength were respectively set to 4, 512, and 0.05. $\alpha_{0}$ was fixed to 1. *P* was randomly set between 0 and 10, and $\alpha_{p}$ was randomly set between 0 and 0.5. The signal-to-noise ratio (SNR) of synthesized data was also randomly set between 0 dB and 20 dB, where the SNR is defined as $10\log\left(\sigma_{s}^{2}/\sigma^{2}\right)$ [dB].

**Figure 2:**
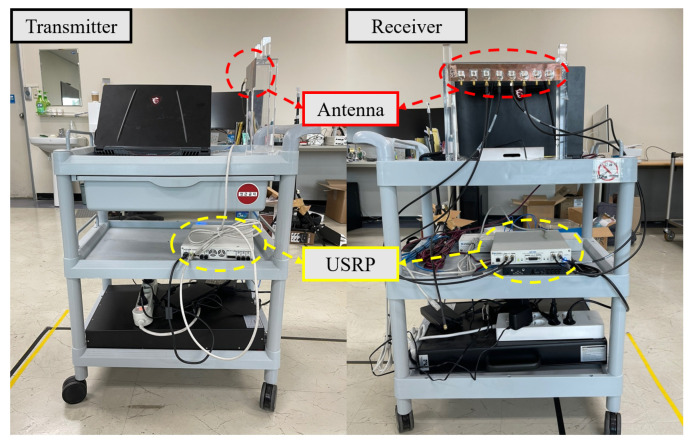
A picture of transmitter and receiver used for the experiment.

Another type of data is that collected with USRP. Note that this data may differ from the signal model in (2). If so, the estimation is expected to be inaccurate when the network is trained with synthesized data. Figure 2 shows a transmitter and a receiver used for the experiment. The transmitter mainly consists of USRP 2954R and the transmitting antenna. The receiver mainly consists of USRP 2955 and a receiving antenna array. Although USRP 2954R and USRP 2955 support a frequency range of 10 MHz–6 GHz, we set the carrier frequency to 5.8 GHz, which is the center frequency of antennas. For this reason, the spacing between patch antennas in the array is designed to the half-wavelength of 5.8 GHz. USRP 2954R generates a 5.8 GHz cosine wave, and the transmitting antenna emits the wave. Then, USRP 2955 receives the cosine wave via the antenna array at a sampling rate of 1 MHz. By using GNU radio, the covariance matrices of the received signals are collected with USRP 2955.

**Figure 3:**
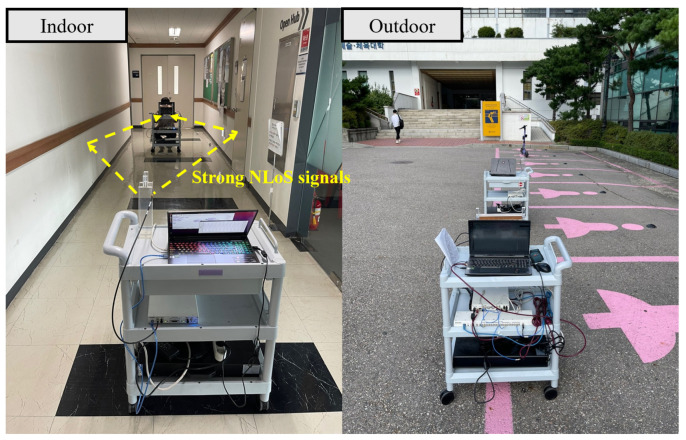
A picture of the indoor hallway and outdoor parking lot. The NLoS signals were expected to be strong in the indoor hallway.

As shown in Figure 3, we data in two different environments, the indoor hallway and the outdoor parking lot. In the indoor hallway, the NLoS signals were expected to be stronger than those in the outdoor parking lot. For this reason, the DoA estimation was expected to be inaccurate in the indoor hallway. DoA estimation range is restricted to [40°,140°] since the radiation pattern of each patch antenna in an array is directional. From 40° to 140° in 10° increments, a total of 17,600 covariance matrices were collected, where half of the data were collected in the indoor hallway while the other half weere collected in the outdoor parking lot. During the experiment, the transmitting power was set to 20 dBm, and the distance between transmitter and receiver was fixed to 6 m. With the collected data, the DoA estimation accuracy is analyzed in the following subsection.

### 4.2. Peformance Analysis and Discussion

Before analyzing the DoA estimation performance, we checked the similarity between collected data and synthesized data. Since the DoAs of the multipath signals weere not measured, we compared the collected covariance matrices with ideal covariance matrices. Ideal covariance matrices are the covariance matrices calculated without considering multipath signals and other hardware-induced errors. The ideal covariance matrix is defined as:

(10) $$R_{\mathrm{ideal}}\left(\theta \right)=a\left(\theta \right)a{\left(\theta \right)}^{H}\in C^{M\times M},\;\theta \in \Theta ,$$

where $R_{\mathrm{ideal}}\left(\theta \right)$ denotes an ideal covariance matrix according to θ. Θ is a set consisting of labeled DoAs of collected data, which equals $\left\{40{}^{\circ},50{}^{\circ},60{}^{\circ},70{}^{\circ},80{}^{\circ},90{}^{\circ},100{}^{\circ},110{}^{\circ},120{}^{\circ},\right.$$\left.130{}^{\circ},140{}^{\circ}\right\}$.

**Figure 4:**
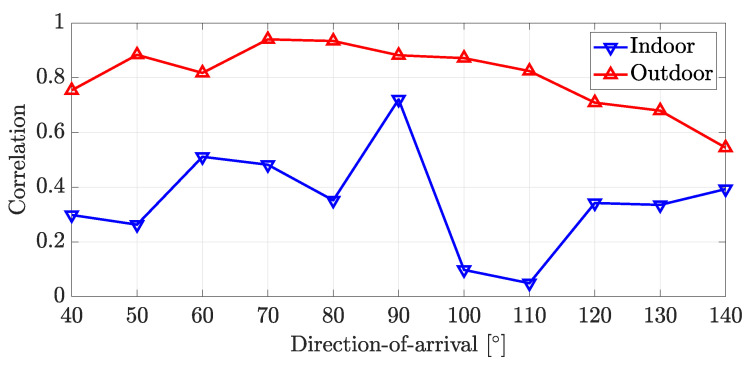
Correlation between collected covariance matrices and ideal covariance matrices according to DoA and experiment environment.

The correlation between collected covariance matrices and ideal covariance matrices is defined as:

(11) $$\rho \left(\theta \right)=E\left[\frac{\langle R_{\mathrm{col}}\left(\theta \right),R_{\mathrm{ideal}}\left(\theta \right)\rangle }{{∥R_{\mathrm{col}}\left(\theta \right)∥}_{F}{∥R_{\mathrm{ideal}}\left(\theta \right)∥}_{F}}\right],\;\theta \in \Theta ,$$

where ρ(θ) denotes the correlation according to DoA. $R_{\mathrm{col}}\left(\theta \right)$ denotes the collected covariance matrix whose DoA label is θ. $\langle A,B\rangle$ denotes the correlation between two matrices, which equals $\mathrm{real}(\mathrm{trace}\left(A^{H}B\right))$. $E\left[\cdot \right]$ denotes the mean calculated using collected data. ρ(θ)∈[0,1], where ρ(θ)=1 when $R_{\mathrm{col}}\left(\theta \right)$ can be represented as $aR_{\mathrm{ideal}}\left(\theta \right)$. Here, *a* is a constant. Figure 4 shows the correlation between collected covariance matrices and ideal covariance matrices according to DoA and the experiment environment. As expected, the data collected in the indoor environment has a low correlation since it suffers from strong multipath signals. On the other hand, the data collected in the outdoor environment has a higher correlation since there are fewer objects that can make multipath signals.

To analyze the DoA estimation performance, we compared five algorithms. One was MUSIC [2]; two were based on DNN and CNN in Section 3.1, and were trained with 4,000,000 synthesized data. The other two algorithms were also based on DNN and CNN in Section 3.1, but they were trained with 75% of the 17,600 collected data points. When using the collected data for training networks, 25% of the 17,600 collected data points were used for testing DoA estimation accuracy. The root mean squared error (RMSE) is defined as $\sqrt{E\left[{\left({\hat{\theta }}_{0}-\theta_{0}\right)}^{2}\right]}$, where $\theta_{0}$ and ${\hat{\theta }}_{0}$ respectively denote the true DoA and the DoA estimated using the test data.

**Figure 5:**
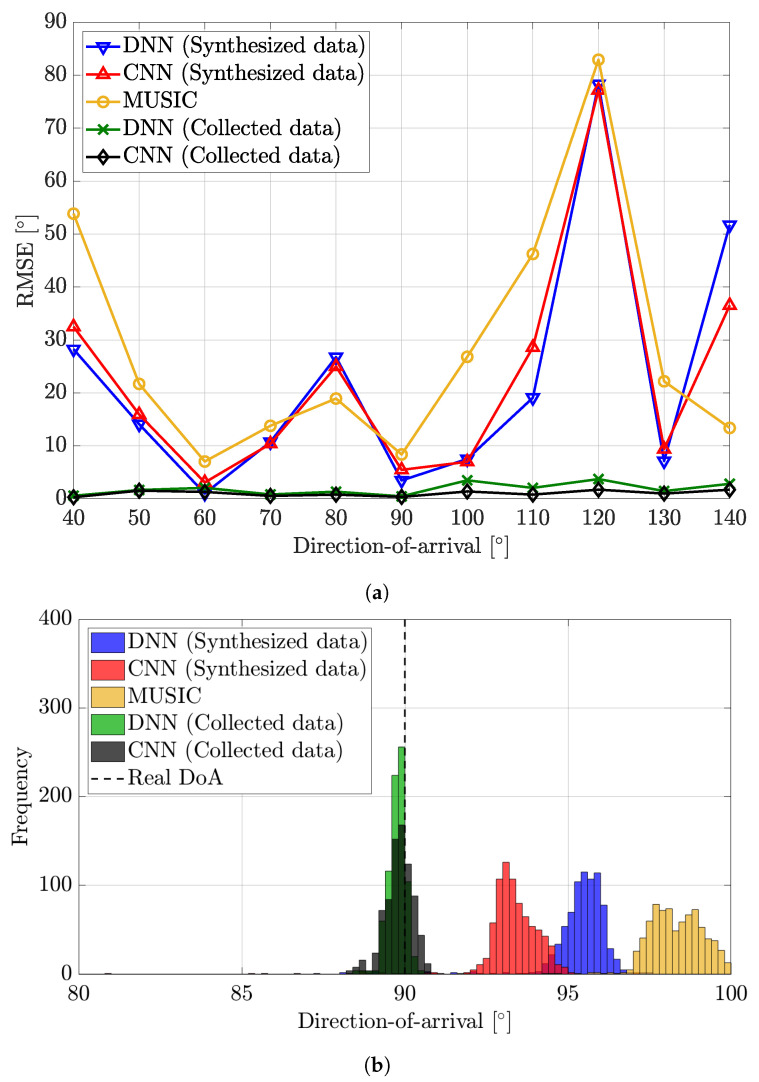
Performance analysis of DoA estimation algorithms in the indoor environment. The first figure shows the RMSE according to DoA, and the second figure is a histogram of estimation results when the actual DoA is 90°. (**a**) RMSE. (**b**) Histogram.

Figure 5 presents two results for the indoor environment, the RMSE of the DoA estimation algorithms and the histogram of estimation results. Both results are derived based on indoor collected data. As expected, Figure 5a,b show that the estimation accuracy is poor in the indoor environment due to the strong NLoS signals. Furthermore, the results also show that the signal model in (2) fails to capture the indoor propagation characteristics since estimation accuracy decreases when using synthesized data for training. However, the DNN and the CNN trained with collected data show much better performance than others. The RMSE of the DNN and the CNN trained with collected data do not surpass 3.5° in every DoA. To be more specific, Figure 5b shows that estimation results tend to gather around the actual DoA when using collected data. When using synthesized data, however, there is a difference between the mean of estimation results and the actual DoA. Moreover, the variance of the estimation is high.

**Figure 6:**
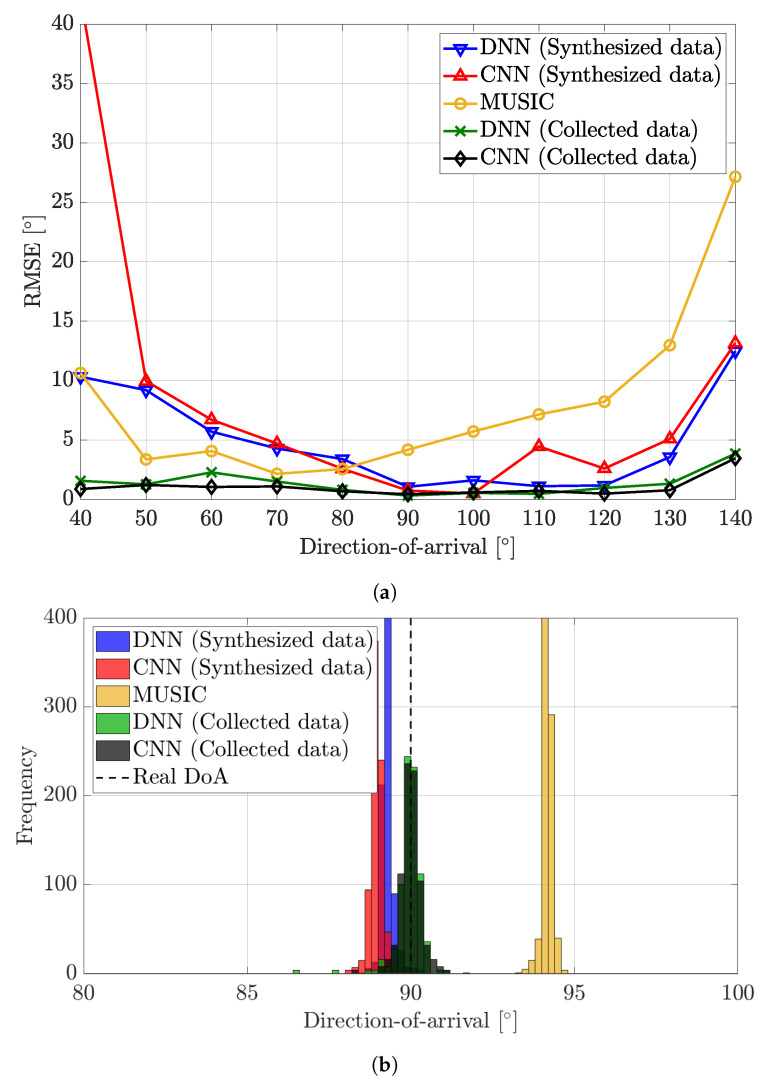
Performance analysis of DoA estimation algorithms in the outdoor environment. The first figure shows the RMSE according to DoA, and the second figure is a histogram of estimation results when the actual DoA is 90°. (**a**) RMSE. (**b**) Histogram.

Figure 6 presents two results for the outdoor environment, the RMSE of the DoA estimation algorithms and the histogram of estimation results. Since the NLoS signals are expected to be much weaker in the outdoor environment, the RMSE of all algorithms are much lower than those in Figure 5a. The RMSE tended to increase when the DoA got far from 90°. We think that this is due to the directivity of the antenna elements. The gain of each antenna element was 5 dBi when the DoA was 90°. However, the gain dropped to 2 dBi when the DoA was 40° or 140°. Overall, the DNN trained with synthesized data were more accurate than MUSIC except in a few DoAs, but the RMSE of the CNN trained with synthesized data was unexpectedly high when the DoA was 40°. Meanwhile, the DNN and the CNN trained with collected data were more accurate than others. To be more specific, when using synthesized data, there was a difference between the mean of estimation results and the actual DoA. However, this difference was smaller than that in Figure 5b. When using collected data, estimation results tended to gather around the actual DoA.

Table 3 shows a total RMSE of all DoA estimation algorithms. In the indoor environment, the algorithms except those using collected data showed poor performance. Among them, the DNN and CNN trained with synthesized data showed slightly better performance than MUSIC. Although the performance of all algorithms improved in the outdoor, the DNN and CNN trained collected data showed much better performance than others. Since the RMSE of the CNN trained with synthesized data soared when the DoA was 40°—its total RMSE was larger than that of MUSIC.

Table 4 shows the training time, computation time, and computational complexity of each algorithm. Here, the computational complexity of CNN is derived using [28]. The training time is proportional to the amount of training data. When training a network with 4,000,000 synthesized data points, it took 3500 and 4400 seconds to train the DNN and CNN. On the other hand, it took 190 and 230 seconds to train the DNN and CNN when using 13,200 collected data points. Although the DNN and CNN-based DoA estimation take a long time for training, their computational complexity is much less than MUSIC once the networks are trained.

From all results, we conclude that training with collected data enables accurate DoA estimation. However, collecting sufficient data can be difficult in practice. One of the solutions to this problem is using the synthesized data that well capture the characteristics of the realistic wave. Another solution is to use unsupervised learning such as [20]. Unsupervised learning can make collecting data much easier since data labeling is not required.

## 5. Conclusions

We present the experimental validation of the deep learning-based DoA estimation using USRP. The DNN and the CNN structures are designed to estimate the DoA of the LoS path with the covariance matrix. In the experiment, two types of data are exploited. One is the data synthesized with the signal model, and the other is the data collected by USRP. The experimental results show that the DoA estimation is most accurate when training DNN and CNN with the collected data. Furthermore, the DoA estimation performance is poor in the indoor environment, which suffers from the strong NLoS signals. However, collecting sufficient data may not be feasible in practice. We expect that this can be resolved by better signal modeling and unsupervised learning.

## Figures and Tables

**Table 1:** Parameter setting for DNN structure.

Parameter	Value (or Type)
Number of layers (*L*)	2
Size of layers ($I^{\left(l\right)}$ for l=1,…,L)	600, 600
Loss function	MSE
Optimizer	Adam
Activation function	ReLU
Batch size	100

**Table 2:** Parameter setting for CNN structure.

Parameter	Value (or Type)
Number of layers (*K*)	3
Number of kernels (J(k) for k=1,…,K)	50,150,300
Size of kernels ($Q_{k}$ for k=1,…,K)	3,2,1
Loss function	MSE
Optimizer	Adam
Activation function	ReLU
Batch size	100

**Table 3:** A total RMSE of DoA estimation algorithms.

	DNN (Synthesized Data)	CNN (Synthesized Data)	MUSIC	DNN (Collected Data)	CNN (Collected Data)
**Indoor**	31.8°	30.6°	36.2°	2.1°	1.2°
**Outdoor**	6.2°	14.1°	10.6°	1.6°	1.3°

**Table 4:** Analysis on training time, computation time, and computational complexity.

	Training Time [s]	Computation Time [μs]	Computational Complexity
**DNN (Synthesized data)**	3500	33	$O\left(\left(M^{2}+I^{\left(2\right)}\right)I^{\left(1\right)}\right)$
**DNN (Collected data)**	190		
**CNN (Synthesized data)**	4400	35	$O\left(M^{2}\sum_{i=2}^{3}Q_{i}^{2}J\left(i-1\right)J\left(i\right)\right)$
**CNN (Collected data)**	230		
**MUSIC**	-	7161	$O\left(M^{3}\right)$

## Data Availability

Not applicable.

## Abbreviations

The following abbreviations are used in this manuscript:

DoA	Direction-of-arrival
USRP	Universal software radio peripheral
DNN	Deep neural network
CNN	Convolution neural network
RNN	Recurrent neural network
ResNet	Residual neural network
MLE	Maximum likelihood estimation
LoS	Line-of-sight
NLoS	Non-line-of-sight
MUSIC	Multiple signal classification
ESPRIT	Estimation of signal parameters via rotational invariance techniques
ULA	Uniform linear array
MSE	Mean squared error
SNR	Signal-to-noise ratio
RMSE	Root mean squared error
